# Weighted Fused Pathway Graphical Lasso for Joint Estimation of Multiple Gene Networks

**DOI:** 10.3389/fgene.2019.00623

**Published:** 2019-07-22

**Authors:** Nuosi Wu, Jiang Huang, Xiao-Fei Zhang, Le Ou-Yang, Shan He, Zexuan Zhu, Weixin Xie

**Affiliations:** ^1^College of Electronics and Information Engineering, Shenzhen University, Shenzhen, China; ^2^College of Computer Science and Software Engineering, Shenzhen University, Shenzhen, China; ^3^School of Mathematics and Statistics, Central China Normal University, Wuhan, China; ^4^Guangdong Key Laboratory of Intelligent Information Processing and Shenzhen Key Laboratory of Media Security, College of Electronics and Information Engineering, Shenzhen University, Shenzhen, China; ^5^Shenzhen Institute of Artificial Intelligence and Robotics for Society, Shenzhen, China; ^6^School of Computer Science, University of Birmingham, Birmingham, United Kingdom

**Keywords:** Gaussian graphical model, precision matrix, prior information, fused lasso penalty, gene network analysis

## Abstract

Gene regulatory networks (GRNs) are often inferred based on Gaussian graphical models that could identify the conditional dependence among genes by estimating the corresponding precision matrix. Classical Gaussian graphical models are usually designed for single network estimation and ignore existing knowledge such as pathway information. Therefore, they can neither make use of the common information shared by multiple networks, nor can they utilize useful prior information to guide the estimation. In this paper, we propose a new weighted fused pathway graphical lasso (WFPGL) to jointly estimate multiple networks by incorporating prior knowledge derived from known pathways and gene interactions. Based on the assumption that two genes are less likely to be connected if they do not participate together in any pathways, a pathway-based constraint is considered in our model. Moreover, we introduce a weighted fused lasso penalty in our model to take into account prior gene interaction data and common information shared by multiple networks. Our model is optimized based on the alternating direction method of multipliers (ADMM). Experiments on synthetic data demonstrate that our method outperforms other five state-of-the-art graphical models. We then apply our model to two real datasets. Hub genes in our identified state-specific networks show some shared and specific patterns, which indicates the efficiency of our model in revealing the underlying mechanisms of complex diseases.

## Introduction

Most biological processes within cells involve multiple genes (Schlitt and Brazma, 2007; Zhang et al., 2014). Inferring the regulatory relationships between genes is important for understanding the functional organization within cells and helps to reveal the mechanisms of complex diseases (Liu et al., 2016). In recent years, a large number of works have been proposed for inferring gene regulatory networks (GRNs) from gene expression data (Schlitt and Brazma, 2007; Danaher et al., 2014; Verfaillie et al., 2014; Zhang et al., 2014; Liu et al., 2016; Ou-Yang et al., 2017a, Ou-Yang et al., 2017b). Despite their success in addressing some biological problems, revealing the comprehensive GRNs is still a challenging task (Zhang et al., 2014; Grechkin et al., 2015).

Gaussian graphical model (GGM) is an attractive paradigm to depict the associations among biomolecules (Yuan and Lin, 2007). In GGM, each node of the graph represents a random variable from a random vector subjected to multivariate normal distribution, and there is an edge between two nodes if the corresponding two random variables are conditionally dependent, which means the corresponding element of the precision matrix (or inverse covariance matrix) is non-zero (Dempster, 1972; Uhler, 2017). This property makes GGM so popular because we are able to get the network structure by just estimating the precision matrix (Yuan and Lin, 2007). Unfortunately, in the analysis of gene expression data, the number of samples is usually far less than the dimension of a random vector, which makes it hard to estimate the precision matrix directly (the empirical covariance is not invertible). By assuming that the precision matrix is sparse and the data samples are drawn independently from the same distribution, several approaches have been proposed to estimate the precision matrix (Meinshausen and Bühlmann, 2006; Yuan and Lin, 2007; Banerjee et al., 2008).

However, as biological systems are highly dynamic (Ideker and Krogan, 2012), we are faced with observations collected from different states (Huang et al., 2018a). For example, the gene expression data can be collected from both the diseased and normal tissues (Tian et al., 2016; Ou-Yang et al., 2017a). Thus, if we estimate each state-specific network separately for each sample set, the common structures within different state-specific networks will be ignored. In contrast, inferring a single network from all sample sets may mask their differences. To address this problem, many works have been proposed in recent years to jointly estimate multiple graphical models (Guo et al., 2011; Danaher et al., 2014; Ou-Yang et al., 2017b). When the focus is to infer the differential network between two different states, instead of inferring two state-specific networks, some works have also been developed to estimate the differential network directly (Zhang and Zou, 2014; Yuan et al., 2017).

Although the above methods for learning the structure of multiple GGMs have been successfully used to estimate the regulatory relationships among genes, their performance may be limited since they do not consider the existing knowledge about genes and their regulatory relationships. For example, a pathway is a set of components that interact with each other to perform specific biological tasks. Researches have found that many diseases arose from the joint action of multiple genes within a pathway (Peng et al., 2010). Therefore, pathway-based learning of gene regulatory networks may yield biological insights that are hard to detect by traditional GGMs (Grechkin et al., 2015). Although a pathway graphical lasso model has been proposed to incorporate pathway-based constraints into GGMs (Grechkin et al., 2015), it is designed for single network estimation and cannot jointly estimate multiple GGMs. Moreover, with the accumulation of high-throughput data, we are able to collect some literature-curated gene interactions from public database (Han et al., 2017; Xu et al., 2018). As the state-specific gene regulatory relationships as well as their changes across multiple states are more likely to take place between genes that are known to have interactions, incorporating these prior information may help to identify the changes of GRNs more accurately (Xu et al., 2018).

**Figure 1:**
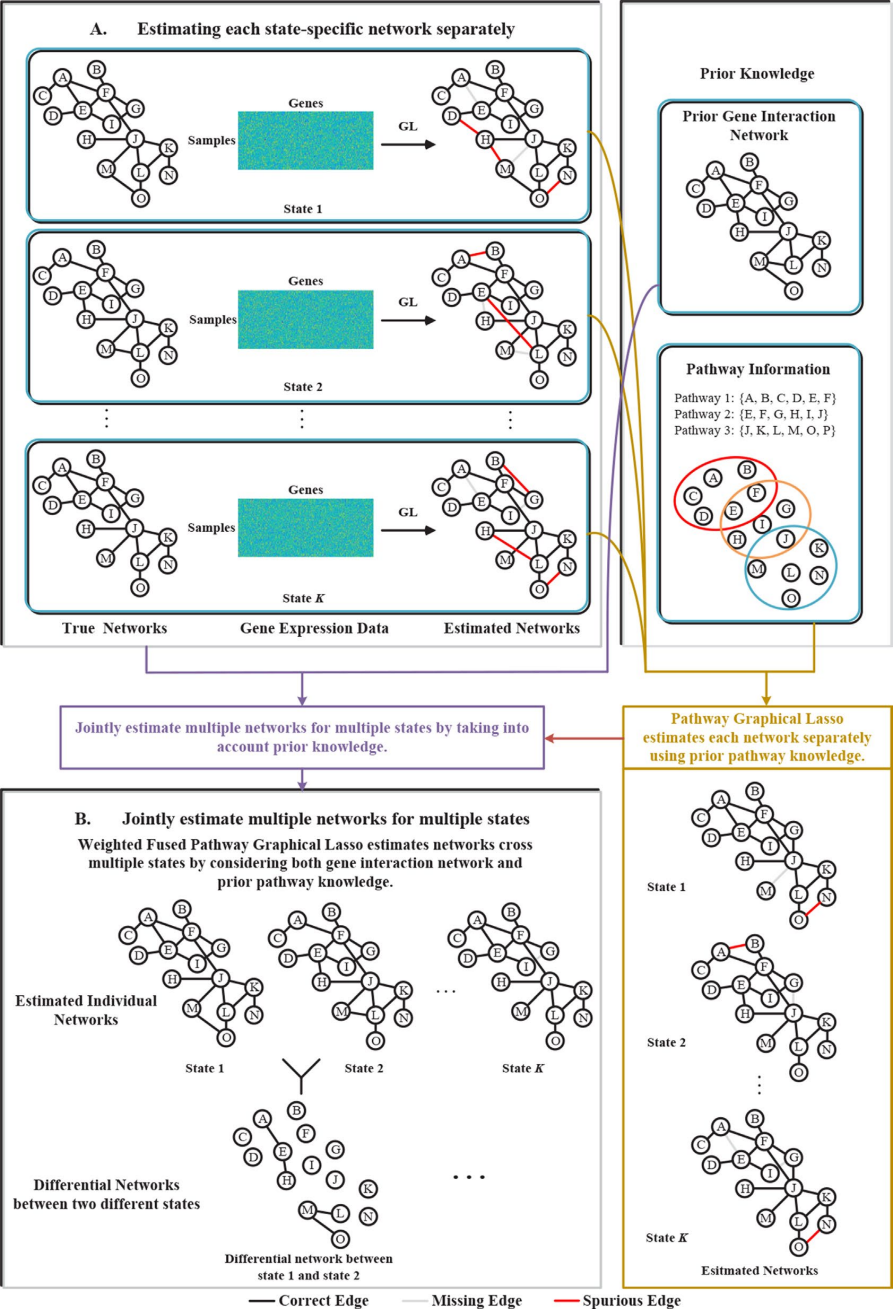
Illustration of the proposed method. **(A)** Graphical lasso just uses gene expression data to separately estimate each state-specific network, leading to incorrect estimation results. **(B)** The proposed weighted fused pathway graphical lasso jointly estimates multiple state-specific networks by considering the prior knowledge of gene interaction networks and pathways, which could eliminate the spurious links between different pathways and results in more accurate estimation of the networks across multiple states.

To address the above problems, in this paper, we proposed a novel weighted fused pathway graphical lasso (WFPGL) to jointly estimate multiple gene networks as well as their difference by incorporating prior knowledge derived from known pathways and gene interactions. In particular, given a set of pathways, we first assume that regulatory relationships will not take place between genes that belong to different pathways. Here, the pathway information is assumed to be able to provide us prior knowledge that certain edges are unlikely to be present (Grechkin et al., 2015). Under this assumption, the incorrect links across pathways will be eliminated, as shown in **Figure 1**. To make use of the prior knowledge from public gene interaction database and draw support from multiple sample sets collected from different states, we introduce a weighted fused lasso penalty in our model. The proposed WFPGL is optimized by alternating direction method of multipliers (ADMM) (Boyd, 2010), and we follow the idea of pathway graphical lasso that decomposes the original problem into pathway-based subproblem to accelerate the optimization. To demonstrate the performance of our new algorithm, we first conduct simulation studies and compare our algorithm with other five state-of-the-art graphical models. Experiment results on synthetic data show that our WFPGL outperforms other related methods. We then apply our WFPGL on two real datasets. The first experiment is to estimate the gene regulatory networks of insulin sensitive and insulin resistant type 2 diabetes patients. Experiment results demonstrate that our method could identify some promising candidate genes related to insulin resistance. The second experiment is to jointly estimate the gene regulatory networks of four breast cancer subtypes. We find that our identified subtype-specific networks have some shared and specific structures, which may help to reveal the mechanisms of cancer differentiation. Overall, the experiment results on synthetic and real data demonstrate that our WFPGL could effectively utilize prior knowledge to jointly estimate multiple gene networks. The datasets and source codes of our proposed WFPGL are freely available at https://github.com/NuosiWu/WFPGL.

The rest of this paper is organized as follows. In the section Weighted Fused Pathway Graphic Lasso, we formulate our WFPGL and describe the optimization processes. In the section Simulation Studies, we illustrate the performance of WFPGL and another five state-of-the-art methods on synthetic datasets. The section Real Data Analysis provides studies on two real datasets, and the section Conclusion discusses the utilization of the method.

## Weighted Fused Pathway Graphical Lasso

### Problem Formulation

Suppose *X* ^(1)^, …, *X* ^(^ *^K^* ^)^ are *K* groups of sample sets that measure the gene expression levels of *p* genes across *K* different states. Each *X* ^(^ *^k^* ^)^ ∈ R*^p^* ^×^ *^n^_k_* may have a different sample size *n_k_*, and samples within each group (or state) are independent and follow same multivariate Gaussian distribution. Then maximizing the likelihood functions of multiple Gaussian graphical model is equivalent to solving the following optimization problem (Uhler, 2017):

(1) $$\underset{\Theta }{\text{maximize}}\sum_{k=1}^{K}n_{k}[\log\{\det(\Theta^{\left(k\right)})\}-\text{tr}(S^{\left(k\right)}\Theta^{\left(k\right)})],$$

where *S^(k)^* is the sample covariance matrix for *X* ^(^ *^k^* ^)^ and Θ^(^ *^k^* ^)^ is the inverse covariance matrix (or precision matrix).

To learn the parameters Θ^(^ *^k^* ^)^ (which represent networks) for *k* = *1, …, K*, additional penalty is often required to guarantee structural recovery especially for high-dimensional setting. Fused lasso penalty, which imposes sparse penalties not only on individual networks but also on the differences between each pair of networks, has been proven to be effective on joint estimation of multiple networks (Danaher et al., 2014). However, traditional fused lasso penalty does not take into account prior information. As the changes of GRNs across different states are more likely to take place between genes that are known to interact with each other (Xu et al., 2018), we encourage the identification of differential edges that appear in the known gene interaction network. Thus, given a prior gene interaction network with adjacency matrix *G*, we introduce the following weighted fused lasso penalty function:

(2) $$F(\{\Theta^{\left(k\right)}\})=\lambda_{1}\sum_{k=1}^{K}\sum_{i\neq j}W_{ij}|\theta_{ij}^{\left(k\right)}|+\lambda_{2}\sum_{k<k^{\prime }}\sum_{i,j}W_{ij}|\theta_{ij}^{\left(k\right)}-\theta_{ij}^{\left(k^{\prime }\right)}|\;.$$

Here, λ_1_ > 0 is a tuning parameter controlling the sparsity of precision matrices, λ_2_ > 0 is a tuning parameter controlling the sparsity of the differential networks, and *W_ij_* is the weight assigned to a pair of genes. In this study, the weight is set as follows:

(3) $$W_{ij}=\{\begin{array}{cc} 1 & if\;G_{ij}=0,\\ w & if\;G_{ij}=1. \end{array}$$

where *w* ∈ [0, 1] is a predefined parameter. The smaller the value of *w*, the more likely the corresponding edge will be identified.

As we could make use of pathway information to specify sets of genes that are more likely to work together, incorporating pathway information may help to improve the structure learning of GRNs and achieve more meaningful and interpretable results. Following the ideal of pathway graphical lasso (Grechkin et al., 2015), we constrain the graphical model so that the elements in precision matrices are fixed to 0 if the corresponding gene pairs are not together in any pathways.

Suppose there are *J* pathways within the *p* genes (denoted as *P_t_*, *t* = 1, …, *J*), and considering the pathway constraints and the knowledge of prior gene interaction network, the objective function of our WFPGL model is as follows:

$$\begin{array}{l} \underset{\left\{\Theta^{\left(k\right)}\right\}}{\text{maximize}}\;\sum_{k=1}^{K}n_{k}[\log\{\det(\Theta^{\left(k\right)})\}-\text{tr}(S^{\left(k\right)}\Theta_{\left(k\right)})]-F(\{\Theta^{\left(k\right)}\})\\ \text{s.t. }\Theta^{\left(k\right)}≽0,\;\theta_{ij}^{\left(k\right)}=0,\text{ if }(i\text{, }j)\notin \overset{J}{\underset{t=1}{\cup }}\{(i,j)|i,j\in P_{t}\}, \end{array}$$

where *F*({Θ^(^ *^k^* ^)^}) takes the form of function (2).

### Optimization

#### Pathway Separable

Given *J* pathways, let $\Theta_{t}^{\left(k\right)}(t=1,\ldots ,J)$ denote a sub-matrix of Θ^(^ *^k^* ^)^, which models the sub-graph within the *p_t_* genes in *t*-th pathway (*p*_1_ + ⋯ + *p_J_* = *p*). After some permutations of the rows and columns under the pathway constraints, Θ^(^ *^k^* ^)^ can be rearranged into the following form:

(5) $$\Theta^{\left(k\right)}=\left[\begin{array}{lll} \Theta_{11}^{\left(k\right)} & \Theta_{12}^{\left(k\right)} & 0\\ \Theta_{21}^{\left(k\right)} & \Theta_{22}^{\left(k\right)} & \Theta_{23}^{\left(k\right)}\\ 0 & \Theta_{32}^{\left(k\right)} & \Theta_{33}^{\left(k\right)} \end{array}\right]$$

where $\Theta_{1}^{\left(k\right)}=\left[\begin{array}{ll} \Theta_{11}^{\left(k\right)} & \Theta_{12}^{\left(k\right)}\\ \Theta_{21}^{\left(k\right)} & \Theta_{22}^{\left(k\right)} \end{array}\right]$ contains all edges in the first pathway, and $\Theta_{other}^{\left(k\right)}=\left[\begin{array}{ll} \Theta_{22}^{\left(k\right)} & \Theta_{23}^{\left(k\right)}\\ \Theta_{32}^{\left(k\right)} & \Theta_{33}^{\left(k\right)} \end{array}\right]$ contains the parameter in the rest of the pathways. $\Theta_{22}^{\left(k\right)}$ is the overlapping part of Θ_1_ and Θ_other_, which is a square matrix containing all links among the genes that co-occur in the pathways as well as the remaining pathways. Applying the Schur complement decomposition, we have

(6) $$\det(\Theta^{\left(k\right)})=\det(\Theta_{33}^{k})\cdot \det(\Theta_{1}^{k}-{∆}_{1}^{\left(k\right)}),$$

where ${∆}_{1}^{\left(k\right)}=[0;\Theta_{23}^{k}]\cdot {\left(\Theta_{33}^{k}\right)}^{-1}\cdot [0,\;\Theta_{32}^{\left(k\right)}]$. If we hold the parameters from other pathways fixed and just update the parameters in the first pathway, ${∆}_{1}^{\left(k\right)}$ will be a constant matrix, and problem (1) boils down to maximizing the following function:

(7) $$n_{k}[\log\{\det(\Theta_{1}^{\left(k\right)})-{∆}_{1}^{\left(k\right)})\}-\text{tr}(S_{1}^{\left(k\right)}(\Theta_{1}^{\left(k\right)}-{∆}_{1}^{\left(k\right)}))],$$

where the matrix with subscript denotes the sub-matrix corresponding to the first pathway. Notice that (7) is exactly the likelihood function for updating $\Theta_{1}^{\left(k\right)}-{∆}_{1}^{\left(k\right)}$, and ${∆}_{1}^{\left(k\right)}$ is constant and can be pre-calculated. Updating $\Theta_{1}^{\left(k\right)}$ within a certain pathway *via* maximizing likelihood function only requires information from the corresponding pathway itself. We define a function with such property to be pathway separable.

#### Optimization Work Flow

Problem (4) is converted into the following equivalent problem:

(8) $$\begin{array}{l} \underset{\left\{\Theta^{\left(k\right)}\},\{Z^{\left(k\right)}\right\}}{\text{minimize}}\;-\sum_{k=1}^{K}n_{k}[\log\{\det(\Theta^{\left(k\right)})\}-\text{tr}(S^{\left(k\right)}\Theta^{\left(k\right)})]+F(\{Z^{\left(k\right)}\}),\\ \text{ s.t. }Z^{\left(k\right)}=\Theta^{\left(k\right)},k=1,2,\ldots ,K, \end{array}$$

which can be solved with the ADMM method (Boyd, 2010) by minimizing the following augmented Lagrangian function:

(9) $$\begin{array}{l} -\sum_{k=1}^{K}n_{k}[\log\{\det(\Theta^{\left(k\right)})\}-\text{tr}(S^{\left(k\right)}\Theta^{\left(k\right)})]+(F\{Z^{\left(k\right)}\})\\ \;+\sum_{k=1}^{K}(\frac{\mu }{2}||\Theta^{\left(k\right)}-Z^{\left(k\right)}|{|}_{F}^{2}\text{+}\langle Y^{\left(k\right)},\Theta^{k}-Z^{\left(k\right)}\rangle ), \end{array}$$

where <*A*, *B*> = tr (*AB^T^*),‖*A*‖ denotes the Frobenius norm of matrix *A*, *μ* > 0 is a penalty parameter, and *Y* ^(^ *^k^* ^)^ is a Lagrangian multiplier. We let *V* ^(^ *^k^* ^)^ = *Y* ^(^ *^k^* ^)^/*μ*, and (9) can be rewritten as the ultimate objective function:

$$\begin{array}{l} L_{\mu }(\{\Theta^{\left(k\right)}\},\{Z^{\left(k\right)}\},V^{\left(k\right)}\})=\\ \;-\sum_{k=1}^{K}n_{k}[\log\{\det(\Theta^{\left(k\right)})\}-\mathrm{tr}(S^{\left(k\right)}\Theta^{\left(k\right)})]+F(\{Z^{\left(k\right)}\})\\ \;+\sum_{k=1}^{K}(\frac{\mu }{2}||\Theta^{\left(k\right)}-Z^{\left(k\right)}+V^{\left(k\right)}|{|}_{F}^{2}-\frac{\mu }{2}||V^{\left(k\right)}|{|}_{F}^{2}). \end{array}$$

**Algorithm 1:** Framework of alternating direction method of multipliers (ADMM).

**Input:** *S*, *n_k_*, *μ*, λ*_1_*, λ*_2_* **Initializing:** *μ* = 1, $\Theta_{0}^{\left(k\right)}=I,V_{\left(0\right)}^{\left(k\right)}=0,Z_{\left(0\right)}^{\left(k\right)}=0,$ for *k* = 1, 2, …, *K*. 1: **while** not converged **do** 2: $\{\Theta_{\left(i\right)}^{\left(k\right)}\}\overset{}{\leftarrow }arg\;min_{\Theta }L_{\mu }(\{\Theta^{\left(k\right)}\},\{Z_{\left(i-1\right)}^{\left(k\right)}\},\{V_{i-1}^{\left(k\right)}\}).$ 3: $\{Z_{\left(i\right)}^{\left(k\right)}\}\overset{}{\leftarrow }arg\;min_{\Theta }L_{\mu }(\{\Theta_{\left(i\right)}^{\left(k\right)}\},\{Z^{\left(k\right)}\},\{V_{i-1}^{\left(k\right)}\}).$ 4: $\{V_{\left(i\right)}^{\left(k\right)}\}\overset{}{\leftarrow }\{V_{\left(i\right)}^{\left(k\right)}\}+\{\Theta_{\left(i\right)}^{\left(k\right)}\}-\{Z_{\left(i\right)}^{\left(k\right)}\}).$ 5: *i* ← *i* + 1 6: **end while**

ADMM solves above problem by three steps in every iteration, as shown in Algorithm 1.

#### ADMM Solver

It is easy to verify that $||\Theta^{\left(k\right)}-Z^{\left(k\right)}+V^{\left(k\right)}|{|}_{F}^{2}$ is pathway separable. This allows us to use a block-coordinate descent approach for accelerating the updating of Θ^(^ *^k^* ^)^ (Tseng, 2001; Grechkin et al., 2015). By updating $\Theta_{t}^{\left(k\right)}$ in each pathway individually while leaving the parameters in other pathway fixed, the complexity of the problem is narrowed down, and the pathway constraints on the precision matrix naturally meet since the parameters outside the pathway remain 0. Specifically, let $T_{t}^{\left(k\right)}=\Theta_{t}^{\left(k\right)}-{∆}_{t}^{\left(k\right)}$, and we update $\Theta_{t}^{\left(k\right)}$ by solving the subproblem, as follows:

$$\begin{array}{l} \underset{\left\{\Theta_{t}^{\left(k\right)}\right\}}{\text{minimize}}\;-n_{k}[\log\{\det(T_{t}^{\left(k\right)})\}-\mathrm{tr}(S_{t}^{\left(k\right)}T_{t}^{\left(k\right)})]\\ +\frac{\mu }{2}||T_{t}^{\left(k\right)}+{∆}_{t}^{\left(k\right)}-Z_{t}^{\left(k\right)}+V_{t}^{\left(k\right)}|{|}_{F}^{2}. \end{array}$$

Here, the subscript *t* of a matrix denotes the index of the sub-matrix that corresponds to the *t*-th pathway. Grechkin et al. (2015) have introduced an efficient message passing algorithm to calculate ${∆}_{t}^{\left(k\right)}$ efficiently. The solution to this problem is *UCU^T^*, where *UDU^T^* represents the eigendecomposition of $S_{t}^{\left(k\right)}+\mu /n_{k}({∆}_{t}^{\left(k\right)}-Z_{t}^{\left(k\right)}+V_{t}^{\left(k\right)})$, and *C* is a diagonal matrix with *i*-th diagonal element to be $\frac{n_{k}}{2\mu }\{-D_{ii}+{\left(D_{ii}^{2}+4\mu /n_{k}\right)}^{1/2}\}$

The second step is to update *Z* ^(^ *^k^* ^)^ as the minimizer of

(12) $$\begin{array}{l} \sum_{k=1}^{K}(\frac{\mu }{2}||\Theta^{\left(k\right)}-Z^{\left(k\right)}+V^{\left(k\right)}|{|}_{F}^{2})+\lambda_{1}\sum_{k=1}^{K}\sum_{i\neq j}W_{ij}|z_{ij}^{\left(k\right)}|\\ +\lambda_{2}\sum_{k<k^{\prime }}^{}\sum_{i,j}W_{ij}\text{|}z_{ij}^{\left(k\right)}-z_{ij}^{\left(k^{\prime }\right)}|). \end{array}$$

The solution to this problem can be obtained by the method introduced in Hoefling (2010). The last step for each iteration is to update V^(^ *^k^* ^)^ as V^(^ *^k^* ^)^ + Θ^(^ *^k^* ^)^ − *Z* ^(^ *^k^* ^)^. We stop the algorithm until $\sum_{k}\left|\right|\Theta_{\left(i\right)}^{\left(k\right)}-\Theta_{\left(i-1\right)}^{\left(k\right)}|{|}_{F}^{2}\text{/}\sum_{k}||\Theta_{\left(i-1\right)}^{\left(k\right)}|{|}_{F}^{2}<10^{-5}$

## Simulation Studies

We compare the performance of WFPGL with that of five state-of-the-art graphical models: 1) graphical lasso (GL) (Meinshausen and Bühlmann, 2006), which is a classical algorithm for precision matrix estimation; 2) pathway graphical lasso (PGL) (Grechkin et al., 2015), which is a framework that uses pathway knowledge to estimate single Gaussian graphical model; 3) fused graphical lasso (FGL) (Danaher et al., 2014), which is a method for joint estimation of multiple precision matrices across multiple states; 4) differential network estimation *via* D-trace loss (Dtrace) (Yuan et al., 2017), which is a method for direct estimation of a differential network between two states; and 5) weighted D-trace loss (WDtrace) (Xu et al., 2018), which is an algorithm proposed for inferring differential network rewiring by integrating static gene regulatory network information. We implement GL and FGL with their R packages and perform PGL and WFPGL in python environment and using Matlab to carry out Dtrace and WDtrace.

WFPGL has three tuning parameters, i.e., the sparsity controllers λ_1_ and λ_2_ and the weight of prior information *w*. For *w*, as suggested by Xu et al. (2018), we set *w* = 0.3 in the following experiments.

To evaluate the performance of various, we adopt four evaluation metrics named true-positive rate (TPR), false-positive rate (FPR), true-positive differential rate (TPDR), and false-positive differential rate (FPDR). Let *θ_ij_* and ${\hat{\theta }}_{ij}$ denote the elements in true precision matrix and the estimated precision matrix, respectively. The definitions of these four metrics are as follows:

$$\begin{array}{c} TPR=\frac{\sum_{k=1}^{K}\sum_{i<j}I\{{\hat{\theta }}_{ij}^{\left(k\right)}\neq 0\text{ and }\theta_{ij}^{\left(k\right)}\neq 0\}}{\sum_{k=1}^{K}\sum_{i<j}I\{\theta_{ij}^{\left(k\right)}\neq 0\}},\\ FPR=\frac{\sum_{k=1}^{K}\sum_{i<j}I\{{\hat{\theta }}_{ij}^{\left(k\right)}\neq 0\text{ and }\theta_{ij}^{\left(k\right)}=0\}}{\sum_{k=1}^{K}\sum_{i<j}I\{\theta_{ij}^{\left(k\right)}=0\}},\\ TPDR=\frac{\sum_{k<k^{\prime }}\sum_{k<j}I\{{\hat{\theta }}_{ij}^{\left(k\right)}\neq {\hat{\theta }}_{ij}^{\left(k^{\prime }\right)}\text{ and }\theta_{ij}^{\left(k\right)}\neq \theta_{ij}^{\left(k^{\prime }\right)}\}}{\sum_{k<k^{\prime }}\sum_{i<j}I\{\theta_{ij}^{\left(k\right)}\neq \theta_{ij}^{\left(k^{\prime }\right)}\}},\\ FPDR=\frac{\sum_{k<k^{\prime }}\sum_{i<j}I\{{\hat{\theta }}_{ij}^{\left(k\right)}\neq {\hat{\theta }}_{ij}^{\left(k^{\prime }\right)}\text{ and }\theta_{ij}^{\left(k\right)}=\theta_{ij}^{\left(k^{\prime }\right)}\}}{\sum_{k<k^{\prime }}\sum_{i<j}I\{\theta_{ij}^{\left(k\right)}=\theta_{ij}^{\left(k^{\prime }\right)}\}}, \end{array}$$

According to the above definitions, TPR and FPR measure the accuracy of network estimation, whereas TPDR and FPDR measure the accuracy of differential network estimation.

### Experiments on Two Groups of Samples

In this section, we first consider the situation when there are two groups of samples corresponding to two different states. The main generation procedure of the synthetic datasets is described as follows.

STEP 1: PATHWAY DEFINITION
  For ease of simulation study, we simply put successive features into one pathway and let the intersection of two pathways to be non-empty set only when the pathways are “neighbors.” We create 10 pathways with same size that covers all 400 features with *n*_ol_ = 5 features overlapped in neighbor pathway. Let *P_t_* (*t* = 1, …, 10) represent a pathway set in which the element indicates the index of feature. In such configuration, pathways are generated as *P*_1_ = {1, 2, …, 45}, *P*_2_ = (41, 42, …, 85}, …, *P*_10_ = (361, 362, …, 400}.
STEP 2: NETWORK CONSTRUCTION
  We first build a random scale-free network for the state 1, denoted by its adjacency binary matrix *M* ^(1)^. ${\tilde{M}}^{\left(1\right)}$ is a copy of *M* ^(1)^ with each non-zero element substituted by a uniform distribution value on [−0.6, −0.3] ∪ [0.3, 0.6]. Then ${\tilde{M}}^{\left(2\right)}$ is generated by the copy of ${\tilde{M}}^{\left(1\right)}$, with about *r* = 30% non-zero elements vanished.
STEP 3: PRIOR NETWORK GENERATION
  We select a proportion (ㅰ*η*) of edges from *M* ^(1)^ and connect the corresponding features in prior gene interaction network *G*.
STEP 4: PRECISION MATRIX CALCULATION
  To ensure the positive definiteness of the covariance matrix, we get the real precision matrix ${\tilde{\Theta }}^{\left(k\right)}$ as

$${\tilde{\Theta }}^{\left(k\right)}={\tilde{M}}^{\left(k\right)}\circ Q+\sigma I,$$

where ⚬ is Hadamard product operator, *Q* is a *p* × *p* binary “pathway network” matrix in which the “1” element indicates the corresponding feature pairs that co-occur in any of the pathways, σ is the absolute value of the minimum eigenvalues of $\left({\tilde{M}}^{\left(k\right)}\circ Q\right)$ for *k* = 1, 2, and *I* is identity matrix.

After the precision matrix ${\tilde{\Theta }}^{\left(k\right)}$ for each state is settled in Step 4, the synthetic gene expression data could be generated with zero means and $\Sigma ={\tilde{\Theta }}^{-1}$.

We generate 50 random two-state datasets with the same setting: *n_k_* = 100 samples for both two states, 10 pathways with an overlapping number *n*_ol_ = 5 covering all *p* = 400 genes as introduced in Step 1.

As defined in Step 3 of dataset generation, prior rate *η* controls the proportion of true edges that is covered by prior gene interaction network. Note that the edges in the prior gene interaction network are not necessarily differential edges. We set *η* to be 0, 0.4, and 0.8 to see its impact on the performance of WFPGL.

**Figure 2:**
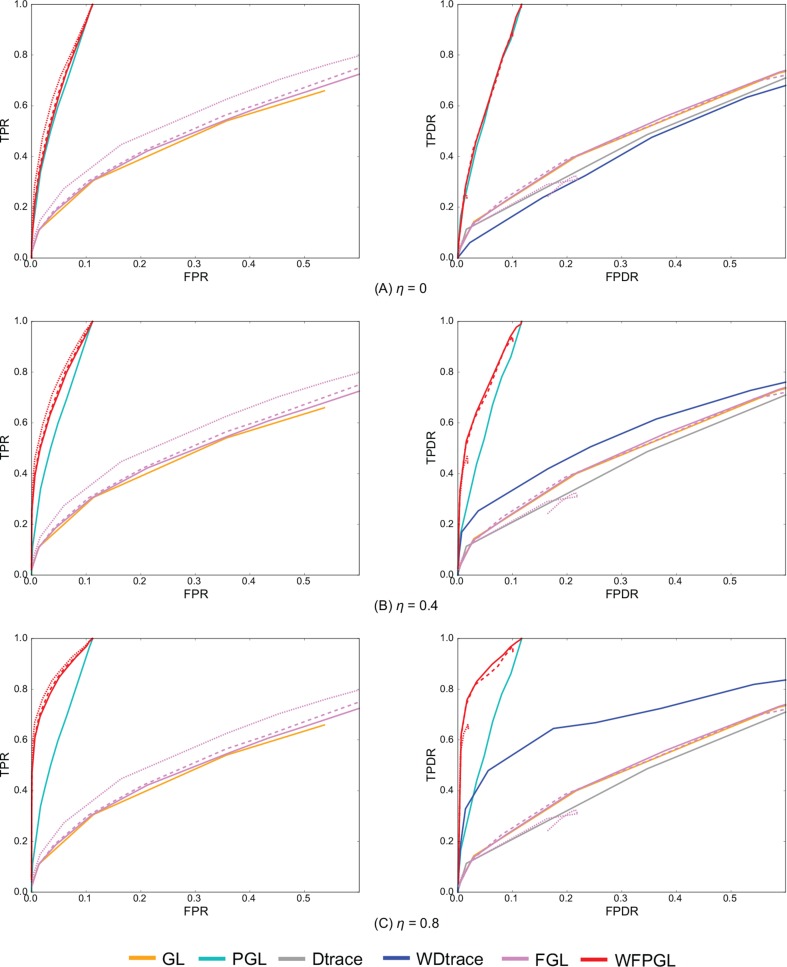
The experiment results of various methods on two groups of samples, with the value of η changing at **(A)** η = 0, **(B)** η = 0.4, and **(C)** η = 0.8. The performance of various methods on individual network estimation [with respect to true-positive rate (TPR) and false-positive rate (FPR)] is shown on the left side, while the performance of various methods on differential network estimation [with respect to true-positive differential rate (TPDR) and false-positive differential rate (FPDR)] is shown on the right side. For weighted fused pathway graphical lasso (WFPGL) and fused graphical lasso (FGL), different line styles correspond to different choices of λ_2_: solid line for λ_2_ = 0.0001, dashed line for λ_2_ = 0.001, and dotted line for λ_2_ = 0.01.

**Figure 2** presents the average performance of various methods over 50 random generations of data with different values of parameters. In particular, for GL, PGL, Dtrace, and WDtrace that have one sparsity-controlling parameter, we vary the value of the parameter and show their performance. For FGL and WFPGL that have two tuning parameters, i.e., λ_1_ and λ_2_, which control the sparsity of individual networks and their difference, respectively, we fix the value of λ_2_ and show their performance with the value of λ_1_ varied. The performance of different methods is shown in distinct colors. Dtrace and WDtrace do not have TPR and FPR, since these two methods directly estimate the differential network between two states and do not predict the individual networks. For GL, PGL, Dtrace, and WDtrace, each curve depicts the performance from a wide range of the sparsity-controlling parameter. For FGL and WFPGL, we pick up three curves here to show their performance. Each curve presents the results with λ_2_ being fixed to a certain value: solid line for λ_2_ = 0.0001, dashed line for λ_2_ = 0.001, and dotted line for λ_2_ = 0.01.

The plots on the left side of **Figure 2** show the performance of various methods for individual network estimation. We can find that pathway-based methods WFPGL and PGL dominate other methods that do not use pathway information. Joint estimation methods outperform single network estimation methods (WFPGL performs better than PGL, and FGL performs better than GL). This may be because the joint estimation methods can draw support from multiple sample sets to achieve a more accurate estimation. Though λ_2_ controls the sparsity of differential networks, it also affects the accuracy of individual network estimation with larger values result in a better prediction.

The plots on the right side of **Figure 2** show the results of differential network prediction. WFPGL also outperforms other methods, since it uses both two views of prior knowledge, followed by PGL that only uses pathway information and WDtrace that only uses prior gene interaction network knowledge. GL, FGL, and Dtrace, which do not use any prior knowledge, cannot identify differential networks accurately. There is a distinct improvement on the performance of WFPGL and WDtrace when the value of *η* increases, which indicates the effectiveness of using prior gene interactions.

To evaluate the performance of various methods without prior network information or prior pathway information, we show the results of various methods without prior network information (i.e., *η *= 0) in **Figure 2**, and we show the results of various methods without prior pathway information in the **Supplementary Material (Figure S1)**. In fact, when there is no prior network information, our method degenerates to a fused pathway graphical lasso model. When there is no prior pathway information, our method degenerates to a weighted fused graphical lasso model. We can find from **Figure 2** and **Figure S1** that when there is no prior network or prior pathway information, our method could still achieve competitive performance with other comparative methods.

### Experiments on Three and Four Groups of Samples

We generate three groups of samples (corresponding to three different states) with *p* = 400 features and *n* = 50 samples per group, and we assume that there are eight pathways covering all features with the overlapping *n_ol_* = 10. The first two networks are generated in the same way as the above section, except that only *r* = 10% edges are deleted from state 1 to form the network of state 2 (Step 2). The precision matrix for state 3 is copied from state 2, with the removed elements in state 2 being reassigned by uniform distributed values [−0.6, −0.3] ∪ [0.3, 0.6] with the possibility of 0.5.

To further test the robustness of WFPGL, we generated a dataset with four different states. In particular, we generated four groups of samples with *p* = 200 features and *n* = 50 samples per group, covered by five pathways with *n_ov_* = 8. The first three networks are generated the same way as the above section except that the different rate is set to be *r* = 20%. Then we randomly deleted 20% non-zero elements from state 1 to build the network of state 4. 

**Figure 3:**
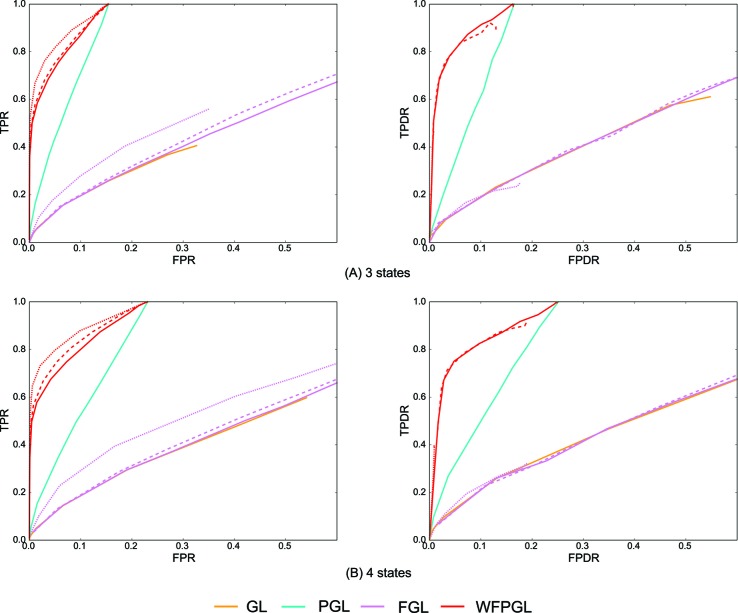
The experiment results of various methods on multiple groups of samples: **(A)** Dataset 1 for three states. **(B)** Dataset 2 for four states. The performance of various methods on individual network estimation (with respect to TPR and FPR) is shown on the left side, while the performance of various methods on differential network estimation (with respect to TPDR and FPDR) is shown on the right side. For WFPGL and FGL, different line styles corresponding to different choices of λ_2_: solid line for λ_2_ = 0.0001, dashed line for λ_2_ = 0.001, and dotted line for λ_2_ = 0.01.

Similarly, 50 random datasets are generated for the above two situations, and **Figure 3** shows the averaged results with *η* = 0.8. Because Dtrace and WDtrace cannot handle the estimation of multiple differential networks, we compare WFPGL with GL, PGL, and FGL. WFPGL still outperforms others on both individual and differential network estimations, followed by PGL, which uses pathway information only. FGL and GL do not perform well on these datasets, though FGL performs slightly better than GL on individual network estimation.

## Real Data Analysis

In this section, we use WFPGL to estimate the gene regulatory networks of type 2 diabetes patients and breast cancer patients, with 8,444 known gene interactions from TRRUST database (Han et al., 2017) as prior network information and pathways collected from Kyoto Encyclopedia of Genes and Genomes (KEGG) database (Kanehisa, 2000) as prior pathway information. The tuning parameters (λ_1_, λ_2_) are determined based on the following Akaike information criterion (AIC):

$$\text{AIC}(\lambda_{1}\text{,}\lambda_{2})=\sum_{k=1}^{K}[n_{k}\text{ tr}(S^{\left(k\right)}{\hat{\Theta }}^{\left(k\right)})-n_{k}\log\;\det({\hat{\Theta }}^{\left(k\right)})+2n_{e}^{\left(k\right)}],$$

where ${\hat{\Theta }}^{\left(k\right)}$ is the estimated precision matrix by using the tuning parameters λ_1_ and λ_2_, and $2n_{e}^{\left(k\right)}$ is the number of non-zero entities in ${\hat{\Theta }}^{\left(k\right)}$. We select the parameters λ_1_ and λ_2_, which obtain the outputs that minimize AIC(λ_1_, λ_2_) to generate the final estimation of Θ^(^ *^k^* ^)^.

### Insulin Resistance in Type 2 Diabetes

We downloaded the gene expression data of type 2 diabetes patients from (Elbein et al., 2012), which contain 31 insulin-resistant (IR) samples and 31 insulin-sensitive (IS) samples. Four relevant pathways are used as prior pathway information, i.e., type 2 diabetes mellitus pathway, Wnt signaling pathway, AMPK signaling pathway, and PI3K–AKT signaling pathway. These four pathways are all implicated in type 2 diabetes. Wnt co-receptor (LRP-5) and the Wnt pathway effector TCF7L2 have been revealed in the development of diabetes (Ip et al., 2012). Targeted drugs have been designed to treat type 2 diabetes by activating AMPK signaling pathway (Jeon, 2016) or PI3K–AKT signaling pathway (Huang et al., 2018b).

We normalize each gene to have 0 mean and 1 standard deviation. By merging rows with the same gene name based on their average, 442 genes are covered by the prior pathway information. According to the experiences in simulation studies, we set *w* = 0.3 and choose λ_1_ (ranging from 0.05 to 0.5) and λ_2_ (ranging from 0.1 to 1) based on AIC. Networks for both IR and IS samples are built, and we calculate their difference to identify the network rewiring associated with insulin resistance.

For real data analysis, due to the lack of ground truth, it is hard to know whether the predicted regulatory relationships are real. As the changes of gene regulatory relationships are often derived by the aberrations of certain genes, we assess the performance of our method by quantifying how hub nodes in our estimated differential networks capture known functionally important genes. **Table 1** shows the top 10 hub genes in our predicted differential network between IR and IS patients. Among these genes, overexpression of MYC is verified to prevent insulin resistance (Riu et al., 2003); TP53 is found to be responsible for the formation of tissue-specific insulin resistance (Strycharz et al., 2017). In liver, inactivation of RELA leads to the improvement of insulin sensitivity (Ke et al., 2015); whole-body insulin sensitivity improves when VEGFA is overexpressed in adipose tissue (Fatima et al., 2017); PRKAA2 gene interfering insulin resistance has been observed in the Japanese population (Horikoshi et al., 2006). Although not confirmed, indirect evidence like statistics (Naderpoor et al., 2017) or mouse experiments (Williams et al., 2015) indicate that EGFR and ITGA1 may be good candidates for insulin resistance. Besides, how RAC1 relates to insulin sensitivity after exercise has attracted a lot of attention (Maarbjerg et al., 2011).

**Table 1:** Top 10 nodes with the highest degree in the predicted differential network between insulin-resistant (IR) and insulin-sensitive (IS) patients.

Rank	1	2	3	4	5	6	7	8	9	10
Name	MYC	TP53	RELA	EGFR	NFKB1	RAC1	VEGFA	CREB3L1	ITGA1	PRKAA2

### Breast Cancer Subtypes

We consider the gene expression data from The Cancer Genome Atlas (Cancer Genome Atlas Research Network et al., 2013) that measure the expression levels of 17,327 genes in 511 patients with breast cancer. The observations are classified into four subtypes: 95 for basal-like, 58 for HER2 enriched, and 231 and 127 for luminal A and luminal B, respectively. In this study, we consider seven pathways, namely, apoptosis pathway, hedgehog signaling pathway, homologous recombination pathway, notch signaling pathway, TGF-5 signaling pathway, mTOR signaling pathway, and p53 signaling pathway, which cover 360 genes in the gene expression data. All these pathways are related to breast cancer. Strong evidence shows that reduced apoptosis will cause breast tumor growth, and high levels of apoptosis in a breast tumor are likely to predict worse survival (Parton et al., 2001). DNA repair pathways that targeted genes involved in homologous recombination were discovered to be associated with hereditary breast cancer, while almost 40% of familial and sporadic breast cancers are homologous recombination deficient (den Brok et al., 2017). High notch1 expression was found to be associated with not only high-grade tumors but also poor prognosis for breast cancer (Zardawi et al., 2009). Changes in regulators of p53 activity were demonstrated to be predictive of early relapse in breast cancer (Gasco et al., 2002). mTOR pathway is implicated in endocrine resistance in ER-positive tumors, and the targeted drugs may be used to treat brain metastases (Paplomata and O’Regan, 2014). Dysregulation of hedgehog and TGF-5 signaling pathway have been identified in the development and progression of breast cancer (Habib and O’Shaughnessy, 2016; Skoda et al., 2018).

We build four state-specific networks corresponding to four breast cancer subtypes. We still set *w* = 0.3 but choose λ_1_ ranging from 0.01 to 0.1 and λ_2_ ranging from 0.001 to 0.01. **Table 2** shows the nodes with the highest degree in our inferred networks. Two types of genes are categorized by the fused penalty—cancer-related genes and subtype-specific genes.

**Table 2:** Top 30 nodes with the highest degree in four breast cancer subtypes.

Rank	Basal like	HER2 enriched	Luminal A	Luminal B
1	IGF1	BMP4	PRKACB	BMP4
2	TP53	IGF1	IGF1	IGF1
3	TNF	ID4	BMP4	IFNG
4	BCL2	IFNG	BMP2	FAS
5	FAS	BCL2	TNF	RPS6KB2
6	BMP2	BMP2	RPS6KB2	TP53
7	THBS1	TP53	THBS1	PRKACB
8	PIK3CG	BIRC3	FAS	BAMBI
9	BMP4	RPS6KB1	TP53	THBS1
10	IFNG	FAS	TGFB2	BMP2
11	AKT3	BMPR1B	BIRC3	TNF
12	BAMBI	MYC	BAMBI	PRKAR2B
13	ID4	PIK3CG	BMPR1B	TNFRSF10B
14	CDKN2B	RPS6KB2	RPS6KB1	BMPR1B
15	TGFB2	TNF	AKT3	BMP7
16	INHBB	BMP7	BCL2	ACVR1C
17	RPS6KB2	THBS1	ACVR1C	IL1R1
18	BIRC3	TGFB2	APAF1	BCL2
19	PITX2	INHBB	AKT1	PIK3R1
20	BMP7	DCN	ID4	ID4
21	BMP5	AKT1	LEFTY1	APAF1
22	LEFTY1	PRKACB	FST	BIRC3
23	INHBA	BAMBI	PIK3R1	PITX2
24	MYC	ACVR1C	IFNG	MYC
25	CCNB3	LEFTY1	INHBB	PIK3CD
26	PRKACB	CDKN2A	PRKAR2B	PMAIP1
27	LEFTY2	SMAD9	PIK3CG	DCN
28	BMP6	GADD45A	PIK3R5	AKT1
29	PIK3CD	INHBA	INHBA	INHBB
30	DCN	SERPINB5	PIK3R3	FASLG

The genes appeared in all subtypes are significantly associated with breast cancer. For example, IGF-1 gene is identified as a key player in major signaling pathways involved in breast cancer growth (Christopoulos et al., 2015); TP53 gene mutations have been found in almost all subtypes (Bertheau et al., 2013); TNF gene is highly expressed in breast carcinomas, and its chronic expression supports tumor growth (Kamel et al., 2012); BAMBI and interferon gamma protein (encoded by IFNG gene) are found to inhibit the tumor growth of breast cancer (Shangguan et al., 2012; Ning et al., 2010). In detecting breast cancer, experiments have revealed the diagnostic value of THBS1 protein (Suh et al., 2012). The overexpression and amplification of RPS6KB2 gene as well as FAS gene are reported to be associated with breast cancer prognosis (Pérez-Tenorio et al., 2011; Bębenek et al., 2013); BMP2 is considered as a driving factor for promoting breast cancer stemness, and BMP4 is a potent suppressor of breast cancer metastasis (Huang et al., 2017; Ampuja et al., 2013).

In addition, the genes that emerged only in one subtype are regarded as potential subtype-specific genes, which may have diverse functions across subtypes. In this result, there are three subtype-specific hub genes identified by our method, i.e., CDKN2B in basal-like, and IL1R1 and TNFRSF10B in luminal B. CDKN2B protein is a cyclin-dependent kinase inhibitor that functions as a cell growth regulator, and its methylation is part of triple negative breast cancer (TNBC) profile (Branham et al., 2012). This result is also in accordance with a recent study (Bareche et al., 2018) that declared that the copy number aberrations of CDKN2B gene suffer a high gain in basal-like 1 subtype. The high expression of IL-1a has been found to be correlated with better prognosis in luminal B breast cancer (Dagenais et al., 2017). Since IL1R1 protein is the receptor for IL-1α, there may be a latent relationship between the IL1R1 gene expression level and luminal B breast cancer. TNFRSF10B (also named as DR5) is a cell surface receptor that can be activated by tumor necrosis factor-related apoptosis inducing ligand (TNFSF10/TRAIL) (Benson et al., 2018). TRAIL kills tumor cells while sparing normal cells and becomes a drug target. However, TRAIL may be selective to patients, since only a small subset of patients respond well to TRAIL in previous clinical trials (Dimberg et al., 2013). Considering that the lack of surface DR5 is sufficient to render tumors resistant to the targeted therapies (Twomey et al., 2015), the correlation between DR5 and luminal B discovered by this paper could provide a new insight of TRAIL therapies on their receivers.

## Conclusion

In this paper, we propose a novel weighted fused pathway graphical lasso (WFPGL) that can effectively incorporate additional knowledge including pathway information and gene interaction networks to jointly estimate multiple gene regulatory networks. These two kinds of prior information have different effects on our algorithm. We incorporate gene interaction priors by assigning a weight matrix to the estimated individual networks and differential networks. When there is no prior gene interaction information, all elements in the weight matrix are set to 1, and our model degenerates to a fused pathway graphical lasso model. For prior pathway information, we utilize the information by imposing constraints that a pair of genes can be connected to each other only if they co-occur in at least one pathway. The constraints have the potential of improving structure learning of gene regulatory networks. First, it can accelerate the optimization of the algorithm, leading to acceptable results when dealing with high-dimensional data. Second, making use of such prior information in learning the structures of networks can yield results that are more meaningful and interpretable. Moreover, as our algorithm is a flexible framework, the “pathway” here does not need to be an exact biological pathway. The “pathway” here stands for a partition of genes such that genes that belong to different “pathways” are less likely to have regulatory relationships. On the one hand, if we can collect additional information, such as the transcript factors (TFs) of genes in a given pathway, we could combine these TFs and their regulated genes into a new “pathway” such that the regulatory relationships are more likely to take place between genes within the same “pathways.” On the other hand, if the pathway information is not comprehensive or remains unknown to us, we could treat all genes as a pathway, and our model degenerates to a weighted fused graphical lasso model.

## Data Availability

Publicly available datasets were analyzed in this study. This data can be found here: https://www.genome.jp/; https://www.ncbi.nlm.nih.gov/geo/query/acc.cgi?acc=GSE40234; https://portal.gdc.cancer.gov/

## Author Contributions

NW conceived and designed the study, performed the statistical analysis, and drafted the manuscript. LO-Y and ZZ conceived the study, and participated in its design and coordination, and helped to draft the manuscript. JH and X-FZ participated in the design of the study, performed the statistical analysis, and helped to revise the manuscript. SH and WX participated in the design of the study and helped to revise the manuscript. All authors read and approved the final manuscript.

## Funding

This work is supported by the National Natural Science Foundation of China under grants No. 61602309, 61871272, 61575125, 11871026 and 61402190, Shenzhen Fundamental Research Program, under grant JCYJ20170817095210760 and JCYJ20170302154328155, Natural Science Foundation of SZU [2017077], Guangdong Special Support Program of Topnotch Young Professionals, under grants 2014TQ01X273, and 2015TQ01R453, Guangdong Foundation of Outstanding Young Teachers in Higher Education Institutions, under grant Yq2015141, Natural Science Foundation of Hubei province [ZRMS2018001337] and Funding of Shenzhen Institute of Artificial Intelligence and Robotics for Society.

## Conflict of Interest Statement

The authors declare that the research was conducted in the absence of any commercial or financial relationships that could be construed as a potential conflict of interest.
