# Supplementary material for: Weighted Fused Pathway Graphical Lasso for Joint Estimation of Multiple Gene Networks
Source: Front Genet. 2019 Jul 22;10:623. doi: 10.3389/fgene.2019.00623 (PMC6662592; doi:10.3389/fgene.2019.00623)
Supplement: Supplementary file 1 [file Presentation_1.pdf]

Supplementary Material:
Weighted Fused Pathway Graphical Lasso for Joint Estimation of Multiple Gene Networks

1 SUPPLEMENTARY FIGURES

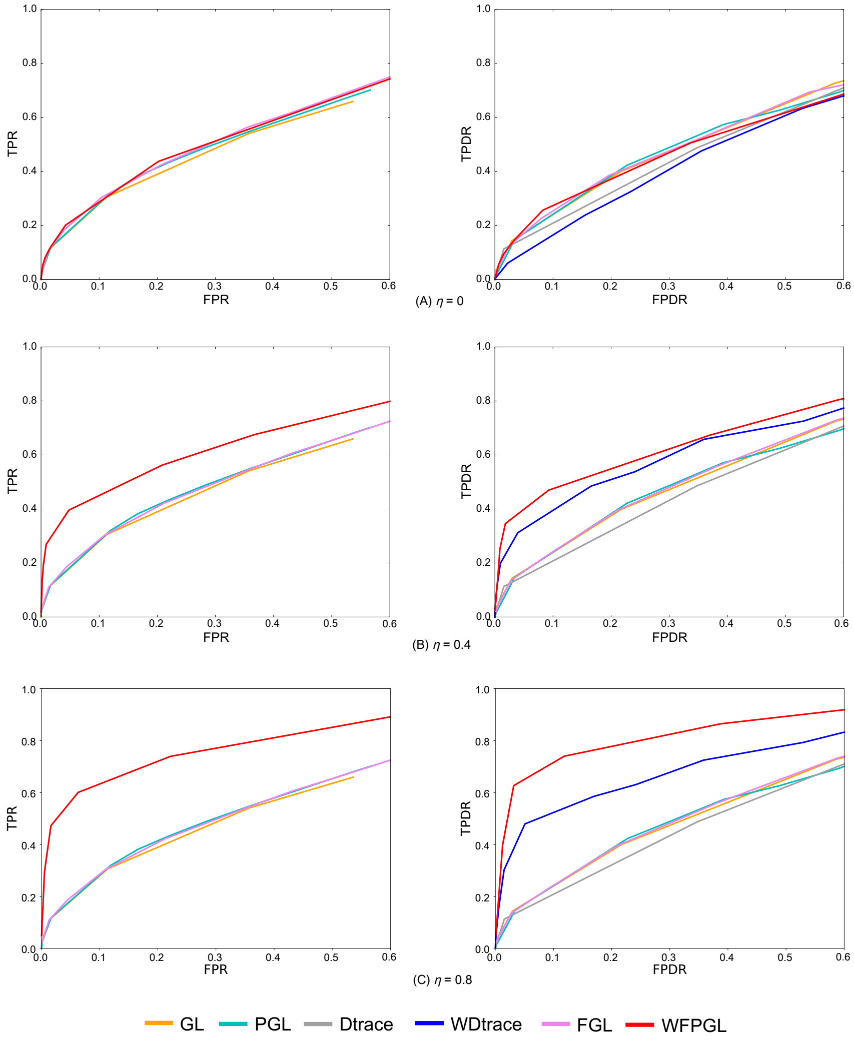

Figure S1. The experiment results of various methods without prior pathway information and the value of η changing from 0, 0.4 and 0.8. The performances of various methods on individual network estimation (with respect to TPR and FPR) are shown on the left side, while the performance of various methods on differential network estimation (with respect to TPDR and FPDR) are shown on the right side. The results of WFPGL and FGL are obtained with $\lambda_2 = 0.0001$.
